# Diagnostic Performance of Point-of-Care High-Sensitivity Troponin in the Exclusion of Non-ST-Elevation Myocardial Infarction in the Emergency Department

**DOI:** 10.3390/jpm14070762

**Published:** 2024-07-17

**Authors:** Daniel Zalama-Sánchez, Carlos del Pozo Vegas, Ancor Sanz-García, Pedro Ángel de Santos-Castro, Javier Presencio-Dominguez, Pablo González-Izquierdo, Susana Sánchez-Ramón, Leyre Teresa Pinilla-Arribas, Manuel Baladrón-Segura, Jaldún Cheayeb-Morán, María Fernandez-García, Guillermo Velasco de Cos, Raúl López-Izquierdo, Francisco Martín-Rodríguez

**Affiliations:** 1 Emergency Department, Hospital Universitario Virgen de la Victoria, 29010 Málaga, Spain; daniel.zalama.sspa@juntadeandalucia.es; 2 Faculty of Medicine, University of Valladolid, 47002 Valladolid, Spain; cpozove@saludcastillayleon.es (C.d.P.V.); rlopeziz@saludcastillayleon.es (R.L.-I.); francisco.martin.rodriguez@uva.es (F.M.-R.); 3 Emergency Department, Hospital Clínico Universitario, 47003 Valladolid, Spain; psantosc@saludcastillayleon.es (P.Á.d.S.-C.); pgonzaleziz@saludcastillayleon.es (P.G.-I.); ltpinilla@saludcastillayleon.es (L.T.P.-A.); jchebhayeb@saludcastillayleon.es (J.C.-M.); 4 Faculty of Health Sciences, University of Castilla la Mancha, 45600 Talavera de la Reina, Spain; 5 Technological Innovation Applied to Health Research Group (ITAS Group), Faculty of Health Sciences, University of Castilla La Mancha, 45004 Toledo, Spain; 6 Evaluación de Cuidados de Salud (ECUSAL), Instituto de Investigación Sanitaria de Castilla-La Mancha (IDISCAM), 45071 Toledo, Spain; 7 Emergency Department, Hospital Universitario Rio Hortega, 47012 Valladolid, Spain; jpresenciod@saludcastillayleon.es (J.P.-D.); ssanchezra@saludcastillayleon.es (S.S.-R.); 8 Clinical Analysis Department, Hospital Universitario Rio Hortega, 47012 Valladolid, Spain; mbaladronsegurab@saludcastillayleon.es (M.B.-S.); mfernandezgarcia1@saludcastillayleon.es (M.F.-G.); 9 Clinical Analysis Department, Hospital Clínico Universitario, 47003 Valladolid, Spain; gvelascod@saludcastillayleon.es; 10 CIBER of Respiratory Diseases (CIBERES), Institute of Health Carlos III, 28029 Madrid, Spain; 11 Advanced Life Support, Emergency Medical Services (SACYL), 47007 Valladolid, Spain

**Keywords:** non-ST-elevation myocardial infarction, troponin, point-of-care testing, emergency department

## Abstract

Background: This study evaluates the diagnostic performance of high-sensitivity troponin using point-of-care testing (POCT) devices compared with main laboratory measurements for ruling out non-ST-elevation myocardial infarction (NSTEMI) in emergency department (ED) patients presenting with non-traumatic chest pain. Methods: This multicenter, observational, prospective, non-interventional study was conducted in two Spanish hospitals from 1 June to 31 December 2023 and included adult patients presenting with non-traumatic chest pain admitted to the ED. High-sensitivity troponin levels were measured using both the Siemens Atellica^®^ VTLi POCT device and main laboratory testing, with data collected on analytical results and measurement times. Results: Of the 201 patients who met the inclusion criteria, a significant correlation was observed between the POCT and laboratory assays. The area under the curve (AUC) of the ROC curve was consistently greater than 0.9, indicating a high diagnostic accuracy for ruling out NSTEMI. In addition, measurement times were significantly reduced using POCT compared to the core laboratory. Conclusion: These results suggest that high-sensitivity troponin POCT devices offer comparable diagnostic performance to traditional laboratory methods for the diagnosis of NSTEMI in the emergency department, potentially speeding up clinical decisions and optimizing resource utilization.

## 1. Introduction

Chest pain is one of the most common reasons for emergency department (ED) visits, accounting for between 5% and 20% of visits. Acute coronary syndrome (ACS) is the most common clinical manifestation of coronary artery disease and the leading cause of morbidity and mortality in Europe. The number of ACS cases is expected to increase in the coming decades, mainly due to the aging population [1]. ACS encompasses several manifestations, including acute myocardial infarction (AMI) and unstable angina. ACS is classified into three categories based on electrocardiographic findings and troponin results: ST-elevation myocardial infarction (STEMI), non-ST-elevation myocardial infarction (NSTEMI), and unstable angina [2]. The final diagnosis of ACS is made by a combination of clinical assessment, ECG analysis and cardiac enzyme measurement. In cases of suspected NSTEMI, the determination of biomarkers, such as troponin, is crucial for diagnosis, risk stratification, and treatment selection [2].

When the clinical presentation is consistent with myocardial ischemia, a troponin (cTn) elevation above the 99th percentile in healthy subjects indicates AMI [3]. In patients with AMI, cardiac troponin levels rise rapidly after symptom onset (usually within the first hour if high-sensitivity assays are used) [2]. The determination of high-sensitivity troponin (hs-cTn) T or I has revolutionized clinical decision making because of its ability to identify very low cTn levels with high diagnostic accuracy and because it is the same cost as low-sensitivity cTn. Therefore, it is currently considered the most recommended test [4,5,6]. Although current algorithms allow AMI to be diagnosed or excluded in most patients within a few hours of their presentation to the emergency department, the time required to obtain the result of the hs-cTn assay in the main laboratory frequently represents a significant obstacle to prompt decision making, primarily due to the necessity of transporting, handling, and processing the samples [7].

The advancement of technology in healthcare has led to the development of numerous tools and instruments designed to assist clinicians in point-of-care (POC) patient assessment. This is particularly beneficial in the ED, where prompt action is of the essence due to the time-dependent nature of many pathologies. A few years ago, point-of-care testing (POCT) devices for cTn measurement were introduced, offering a faster result than those obtained in main laboratories. Nevertheless, they exhibit a lower diagnostic accuracy and a lower negative predictive value [8], which led to the latest European guidelines [2] advising against their use compared to the hs-cTn obtained in main laboratories. However, new devices have recently become available that allow the determination of hs-cTn in less than 10 min, which may change the current recommendations [9].

Therefore, the aim of this study was to evaluate the diagnostic performance of hs-cTn using POCT devices in the exclusion of non-ST-elevation acute coronary syndromes by comparing it with measurements obtained in the main laboratory. In addition, we sought to analyze the ability of these devices to reduce time to action to determine whether they allow earlier clinical decision making.

## 2. Materials and Methods

### 2.1. Study Design and Setting

A prospective, non-interventional, multicenter cohort study was conducted in adult patients presenting with non-traumatic chest pain between 1 August 2023 and 31 December 2023. The study included two university hospitals (Hospital Clínico Universitario de Valladolid (HCUV) and Hospital Universitario Rio Hortega (HURH)) belonging to the public health system of Castilla y León—SACYL—(Spain). The hospitals cover a population of 524,204 inhabitants, are equipped with all medical–surgical services and an intensive care unit (ICU) and treat an average of 145,000 adult emergencies per year. This observational study was conducted after approval by the respective local ethics committees (ref. PI-23-3095 and ref. 23-PI-063) and followed the Strengthening the Reporting of Observational Studies in Epidemiology (STROBE) statement. All participants read and signed the informed consent form.

### 2.2. Participants

The study examined consecutive adult patients (age ≥ 18 years) who presented with non-traumatic chest pain in the ED. The study excluded patients with acute coronary syndrome with ST-segment elevation, advanced chronic kidney disease on dialysis, pregnant women, cases in which analytical determinations could not be made, and those who did not provide informed consent.

### 2.3. Outcome

Diagnosis of NSTEMI by hs-cTn POCT compared with main laboratory determination: the diagnosis of NSTEMI was based on the universal definition of AMI [3], which requires evidence of a troponin rise or fall of at least one concentration above the sex-specific 99th percentile, together with evidence of myocardial ischemia (ischemic symptoms, ECG changes, or imaging tests).

### 2.4. Study Protocol and Data Collection

Patients who met the inclusion criteria underwent hs-cTn measurement using a POCT device and in the main laboratory at two time points during their care: on admission to the ED and 3 h after the initial measurement. This procedure tested for ACS at both study centers.

The Siemens Atellica^®^ VTLi hs-cTn POCT (Siemens Healthcare GmbH Henkestr. 127 91052 Erlangen, Germany) assay was performed in accordance with the standard clinical practice protocol for suspected NSTEMI, which meets the high-sensitivity designation based on established analytical performance criteria. Sex-specific 99th percentile upper reference limits were used (27 ng/L for male patients and 18 ng/L for female patients, with coefficients of variation ranging from 7.1% to 9.5% between 12.2 and 14.0 ng/L) [10]. The device uses a disposable cartridge into which the fresh lithium-heparinised whole blood sample is placed and performs troponin I determination using an immunoassay analyzer. Medical and nursing staff in the emergency departments of both hospitals were trained in the correct use of the device. A uniform system for collecting blood samples from each patient was established. A single venipuncture provided one sample to be sent to the main laboratory, while another with lithium heparin was used for POCT analysis. Once the disposable cartridge had been inserted into the POCT device and the patient identification number entered, the sample was analyzed in approximately eight minutes. The time and test result were recorded.

At the HCUV, main laboratory analysis was performed using the Roche Elecsys^®^ (Roche diagnostics Basel, Switzerland) high-sensitivity troponin T assay, which has a 99th percentile upper reference limit of 14 ng/L with a corresponding coefficient of variation of 10% at 13 ng/L [11]. At the HURH, analysis was performed in the main laboratory using the Beckman Coulter Access hsTnI high-sensitivity troponin I assay, which has a 99th percentile upper reference limit of 17.5 ng/L with a 95% confidence interval of 12.6–20.7 ng/L [12].

Information of 17 comorbidities to calculate the adjusted-age Charlson comorbidity index (aCCI) (myocardial infarction, congestive heart failure, peripheral vascular disease, stroke or transient ischemic attack, dementia, chronic obstructive pulmonary disease, connective tissue disease, peptic ulcer disease, mild liver disease, uncomplicated diabetes mellitus, hemiplegia, moderate to severe chronic kidney disease, diabetes mellitus with end-organ damage, localized solid tumor, leukemia, lymphoma, moderate to severe liver disease, metastatic solid tumor, and acquired immunodeficiency syndrome), test results, ED discharge diagnosis, and the need for hospitalization and intensive care unit (ICU) admissions was collected by reviewing the electronic medical record (EMR). All patient data were recorded electronically in a dedicated database. Access was by individual password and dual authentication.

### 2.5. Data Analysis

Absolute values and percentages were used for the representation of categorical variables and mean and standard deviation for continuous variables. Descriptive results and associations between variables and the result were evaluated using the Mann–Whitney U test or the Chi-squared test, where applicable. The data were collected prospectively and recorded in a database generated with the program IBM SPSS Statistics for Apple version 20.0 (IBM Corp., Armonk, NY, USA). The case registration system was tested to eliminate unclear or ambiguous elements and to verify the suitability of the data collection system. The data present completely random lost values; therefore, the strategy used (removal from the list) does not involve biased mean, variance, or regression weights. The calculation of the sample size yielded the following result: considering a power of 80% and a significance level of 5%, to detect a difference in means of 154 points between pairs and assuming a difference between standard deviations of 436, the minimum sample size per group should be 65. The discriminating power of hs-cTn POCT was evaluated by analyzing the ROC curve and AUC, including 95% confidence intervals (CI) and metrics derived from the ROC curve and AUC. The data were analyzed using our own basic codes and functions (pROC and ROCR packages) in R, version 4.2.2 (http://www.R-project.org (1 November 2022); the R Foundation for Statistical Computing, Vienna, Austria).

## 3. Results

**Figure 1:**
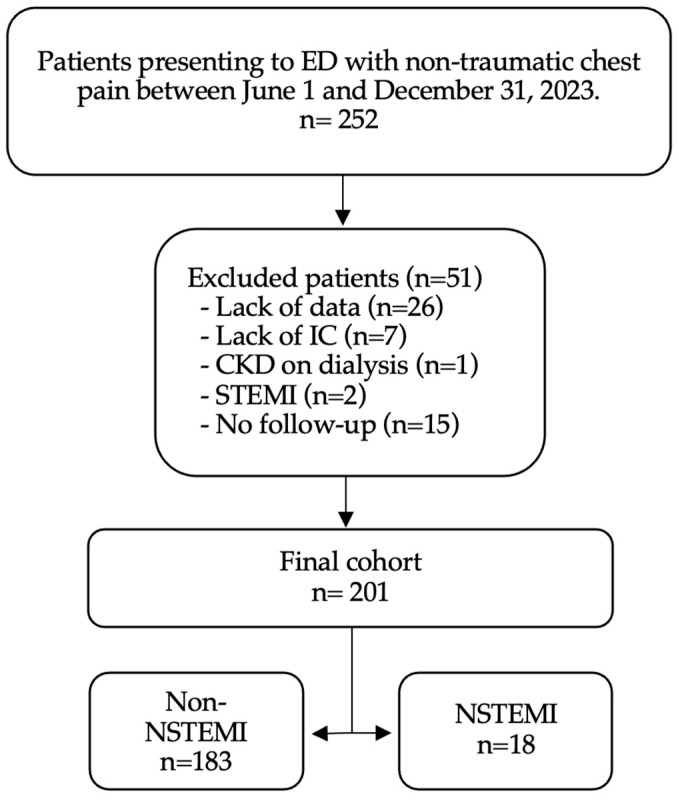
Patient’s flowchart. Abbreviations: CKD: Chronic Kidney Disease, ED: Emergency Department, IC: Informed consent, STEMI: ST-elevation myocardial infraction, NSTEMI: non-ST-elevation myocardial infraction.

A total of 252 patients presenting with non-traumatic chest pain to the ED were initially included in the study. Following the application of exclusion criteria, 51 patients were excluded: 26 due to lack of data, 7 due to lack of informed consent, 1 with chronic kidney disease on dialysis, 2 with STEMI, and 15 without follow-up. Consequently, 201 patients were included in the final analysis, comprising 183 non-NSTEMI and 18 NSTEMI cases (Figure 1).

Patients with a diagnosis of NSTEMI accounted for 9% (n = 18), with a median age of 71 years, and 38.9% (n = 7) were female. They showed ST-segment depression in 66.7% (n = 12), with elevated hs-cTn POCT levels, both at the first measurement with a median of 54.5 ng/L (range: 20.4–463.2) and at the second measurement with a median of 102 ng/L (range: 67.3–443), data that correlated with core laboratory measurements. In patients with NSTEMI, 55.6% (n = 10) were admitted to the intensive care unit and 66.7% (n = 12) underwent percutaneous coronary intervention. Patients without NSTEMI accounted for 91% (183 cases), with a median age of 63 years, 39.9% (n = 73) were female, and only 3.2% (n = 6) had ST-segment depression, while 20.2% (37) had non-specific repolarization abnormalities. In terms of time, hs-cTn POCT provided results 68 min earlier for the first determination in NSTEMI cases compared with the first hs-cTn data in the core laboratory (15 min vs. 83 min), a time saving that was maintained for the second determination (Table 1). In cases without NSTEMI, a time saving of 57 min was achieved for the first measurement (19 min vs. 76 min), which was maintained for the second measurement (Table 1). No differences were found between centers at either the first or second hs-cTn determination (*p* = 0.07 for first POCT hs-cTn, *p* = 0.10 for first main lab hs-cTn, *p* = 0.12 for second POCT hs-cTn, and *p* = 0.18 for second main lab hs-cTn).

The predictive value was assessed by calculating the AUC for each troponin measurement (Table 2), resulting in an AUC of 0.932 (95% CI 0.869–0.995) for the first hs-cTn POCT and an AUC of 0.934 (95% CI 0.889–0.983) for the first hs-cTn in the main laboratory. For the second troponin assay, the AUC was 0.965 (95% CI 0.929–1) for hs-cTn POCT and 0.975 (95% CI 0.953–0.996) for hs-cTn from the main laboratory. In all cases, the AUC was greater than 0.9 and no differences were found between the different measurement methods or acquisition times.

The variables derived from the analysis of the ROC curves and AUC (Table 3) also provided information on their validity, as they were equivalent between the different measurement methods and their acquisition times. For the first hs-cTn POCT, the specificity was 98.83% and the sensitivity was 24.32%, with a positive predictive value (PPV) of 80.73% and a negative predictive value (NPV) of 93.04%. The second hs-cTn POCT demonstrated a specificity of 98.40% and a sensitivity of 23.96%, with a PPV of 65.67% and an NPV of 93.98%. In comparison, the first hs-cTn determination from the central laboratory revealed a specificity of 99.68% and a sensitivity of 14.67%, with a PPV of 98.59% and an NPV of 92.14%. The second central laboratory hs-cTn determination exhibited a specificity of 99.90%, a sensitivity of 7.99%, a PPV of 96.66%, and an NPV of 93.25%.

Finally, the individual measurement times were compared for each acquisition time (Table 4). The median time for the first POCT hs-cTn was 18 min, which was significantly shorter than the 76 min for the first main laboratory hs-cTn. For the second tests, the median time for the POCT hs-cTn was 197 min, compared to 256 min for the main laboratory hs-cTn. In all cases, the difference is statistically significant (*p* < 0.001), with a reduction in time when the measurement was performed using the POCT device. The median time reduction was 54 and 49 min for the POCT method compared to the main laboratory method for the first and second determination, respectively. No differences were found between centers regarding the time saving (*p* = 0.33 for the first time saving and *p* = 0.12 for the second).

## 4. Discussion

This study, which evaluates the diagnostic performance of high-sensitivity troponin using POCT devices (hs-cTn POCT) compared with the main laboratory for the diagnosis of NSTEMI, is notable for being the first to perform hs-cTn POCT in an ED during direct patient care.

The results of the hs-cTn assay using POCT were found to be equivalent to main laboratory determinations in terms of sensitivity, specificity, and both positive and negative predictive values. These data are consistent with the findings of other studies, including those of Apple FS et al. [13], which evaluated the diagnostic accuracy of the Siemens Atellica VTLi hs-cTn POCT for the detection and exclusion of NSTEMI compared with the Abbott ARCHITECT hs-cTn main laboratory assay. The study found a sensitivity of 98.9% and a negative predictive value of 99.5%, with an AUC of 0.85. This was corroborated in an Australian derivation cohort (SAMIE) comparing it to the Beckman Coulter Access hs-cTnI central testing, with comparable results of 98.8% sensitivity, 99.8% negative predictive value, and 0.94 AUC.

Similar studies have also been conducted using alternative POCT hs-cTn measurement devices, resulting in comparable diagnostic capacity. A study [14] utilizing the POCT hs-cTnI PATHFAST device to measure hs-cTn demonstrated a diagnostic capability (AUC = 0.91) comparable to the Abbott Diagnostics ARCHITECT hs-cTn core laboratory test. Another international prospective multicenter study [15] using a POCT device (POCT hs-cTnI TriageTrue) in patients with suspected NSTEMI demonstrated an NPV of 100% with a high diagnostic capability (AUC of 0.95), comparable to two validated core laboratory tests. Additionally, a study performed in a New Zealand ED [16] using the cardiac troponin I POCT device (i-STAT TnI-Nx; Abbott) demonstrated a discriminatory capability comparable to that of the core laboratory to rule out acute myocardial infarction following a single blood test, with an AUC of 0.975.

In contrast to our study, the study by Apple FS et al. [13] indicates that hs-cTn POCT in the ED is performed during the care process by laboratory personnel, rather than ED personnel (physicians and nurses). In the other studies mentioned above [14,15,16], the determination with the POCT device was not performed directly during patient care, but in a delayed manner. The samples were stored and subsequently analyzed with the POCT device in a main laboratory under controlled conditions. Another study similar to ours, conducted at Alkhor Hospital, used a standard POC troponin I assay and found lower sensitivity at first determination. In contrast, our study used a hs-cTn POCT troponin assay and achieved significantly better sensitivity at the first measurement [17].

Another notable aspect of our study is the significant reduction in the time required to obtain hs-cTn values using the POCT device compared with the main laboratory. Our findings indicate a median reduction of 54 min for the initial hs-cTn determination and 49 min for the second determination. These findings are supported by the real-life study by Curran JM et al. [18], which evaluated 50 ED patients with suspected NSTEMI. He found that the Siemens Atellica VTLi POCT device saved an average of 52 min compared to testing performed in the Abbott Alinity core laboratory. The efficiency of obtaining accurate and reliable data in less time with POCT devices could have important implications for early clinical decision making and optimizing resources to improve patient care. This allows for early intervention in cases that require it and excludes patients who do not. Goodacre SW et al. [19], have demonstrated the potential to reduce the length of stay in the ED, as well as the economic costs associated with the use of these tests.

Although diagnostic protocols are becoming faster and demonstrate additional benefits such as reduced ED length of stay and safe discharge [2], the overall implementation rate of these protocols is well below expectations [20]. This may be due to difficulties in meeting the proposed times due to specimen transport issues or increasing ED crowding [20,21]. Blick et al. [22] reported that laboratory turnaround time was a limiting factor in ED time. The use of high-sensitivity troponin POCT could address these difficulties. The use of hs-cTn POCT assays may be the next step to improve the quality and safety of healthcare in the face of increasing demand, while also optimizing triage systems to facilitate rapid decision making and deliver benefits to patients and healthcare systems [23]. A recent study has demonstrated that pre-hospital POC troponin testing, combined with a clinical risk score, can effectively rule out NSTEMI in low-risk patients, significantly reducing costs and the occurrence of major adverse cardiovascular events [24]. Furthermore, the measurement of hs-cTn POCT could facilitate the early assessment of chest pain in the pre-hospital setting [25,26].

Nevertheless, and despite these promising findings, certain limitations of our study must be considered. It is important to keep in mind that the study was performed in a cohort of 201 patients, so additional studies with larger samples are needed to confirm the results obtained. Another limitation to be considered is the variability in troponin measurement between the participating hospitals, using high-sensitivity troponin T in HCUV and high-sensitivity troponin I in HURH. Although both assays are highly validated, this difference may affect the direct comparison of results. Further studies would also be needed to obtain clinical validation in a broader setting to see the impact of decision making on patient outcomes. These future investigations could provide a more detailed perspective on the use of hs-cTn POCT in different clinical settings, thus improving our understanding of its use and implications for medical practice.

In conclusion, this study demonstrated that hs-cTn POCT is equally effective in ruling out NSTEMI, while providing results in a significantly shorter time compared to main laboratory hs-cTn determination.

## Figures and Tables

**Table 1:** Clinical-epidemiological characteristics and follow-up variables.

	Non-NSTEMI	NSTEMI	*p* Value ^b^
No. (%) with data ^a^	183 (91)	18 (9)	
Sex at birth, female	73 (39.9)	7 (38.9)	0.934
Age, year	63 (54–75)	71 (66–83)	0.002
Triage and basal vital signs			
II level	23 (12.6)	9 (50)	Ref.
III level	160 (87.4)	9 (50)	<0.001
Respiratory rate, breaths/min	15 (14–15)	15 (15–17)	0.182
Oxygen saturation, %	97 (95–98)	95 (94–97)	0.060
Systolic blood pressure, mmHg	140 (122–157)	140 (119–151)	0.258
Diastolic blood pressure, mmHg	78 (70–87)	78 (67–88)	0.931
Mean blood pressure, mmHg	97 (89–108)	96 (84–107)	0.4860
Heart rate, beats/min	73 (64–89)	75 (61–87)	0.918
Temperature, °C	36 (35.6–36.3)	36 (35.5–36.2)	0.282
Glasgow coma scale, points	15 (15–15)	15 (15–15)	0.999
Electrocardiogram			
Normal	140 (76.5)	2 (11.1)	Ref.
ST-segment depression	6 (3.2)	12 (66.7)	<0.001
Nonspecific repolarization alterations	37 (20.2)	4 (22.2)	0.026
Time, min			
Triage	10 (6–16)	8 (3–16)	0.178
Support	14 (5–28)	9 (3–17)	0.039
1st POCT hs-cTn	19 (11–30)	15 (11–29)	0.352
2nd POCT hs-cTn	198 (188–212)	194 (188–206)	0.219
1st main lab hs-cTn	76 (63–92)	83 (70–98)	0.459
2nd main lab hs-cTn	257 (235–275)	239 (229–254)	0.263
hs-cTn, ng/L			
1st POCT hs-cTn	4.1 (3.8–8.6)	54.5 (20.4–463.2)	<0.001
2nd POCT hs-cTn	5.6 (3.3–10.6)	102 (67.3–443)	<0.001
1st main lab hs-cTn	5.8 (3.4–12.6)	77.3 (18.8–344.8)	<0.001
2nd main lab hs-cTn	5.8 (3.5–13.1)	142.9 (68–391.9)	<0.001
Outcomes			
Age-adjusted Charlson Comorbidity Index	5 (2–7)	6 (4–10)	0.059
Inpatient	37 (20.2)	18 (100)	<0.001
ICU admission	4 (2.1)	10 (55.6)	<0.001
PCI	16 (8.7)	12 (66.7)	<0.001

Abbreviations: NSTEMI: non-ST-elevation myocardial infarction; Ref: reference; POCT: point-of-care testing; hs-cTn: high-sensitivity cardiac troponin; ICU: intensive care unit; PCI: percutaneous coronary intervention. ^a^ Values expressed as total number (percentage) and medians (25th–75th percentile), as appropriate. ^b^ The Mann–Whitney U test or Chi-squared test was used as appropriate.

**Table 2:** Comparison (Delong’s test) of the discriminating power of troponin by analysis of the ROC curve and the AUC.

Test	1st POCT hs-cTn	1st Main Lab hs-cTn	2nd POCT hs-cTn	2nd Main Lab hs-cTn
1st POCT hs-cTn	0.932 (0.869–0.995)	0.952	0.371	0.209
1st main lab hs-cTn		0.934 (0.886–0.983)	0.320	0.138
2nd POCT hs-cTn			0.965 (0.929–1)	0.653
2nd main lab hs-cTn				0.975 (0.953–0.996)

Abbreviations: POCT: point-of-care testing; hs-cTn: high-sensitivity cardiac troponin.

**Table 3:** Variables derived from the ROC curve and AUC analysis.

		Specificity	Sensitivity	PPV	NPV
	Value	98.83	24.32	80.73	93.04
1st POCT hs-cTn	5% Confidence interval	98.50	23.65	80.13	92.98
	95% Confidence interval	99.16	24.98	81.33	93.10
	Standard error	0.17	0.34	0.31	0.03
					
	Value	99.68	14.67	98.59	92.14
1st main lab hs-cTn	5% Confidence interval	99.56	14.29	98.30	92.11
	95% Confidence interval	99.81	15.06	98.88	92.17
	Standard error	0.06	0.20	0.15	0.02
					
	Value	98.40	23.96	65.67	93.98
2nd POCT hs-cTn	5% Confidence interval	98.05	22.92	65.30	93.90
	95% Confidence interval	98.74	25.01	66.04	94.06
	Standard error	0.18	0.53	0.19	0.04
					
	Value	99.90	7.99	96.66	93.25
2nd main lab hs-cTn	5% Confidence interval	99.89	7.94	96.54	93.24
	95% Confidence interval	99.91	8.03	96.77	93.25
	Standard error	0.00	0.02	0.06	0.00

Abbreviations: POCT: point-of-care testing; hs-cTn: high-sensitivity cardiac troponin; PPV: positive predictive value; NPV: negative predictive value.

**Table 4:** Comparison of hs-cTn determination times (in minutes) for each measurement for each acquisition moment.

	1st POCT hs-cTn	1st Main Lab hs-cTn	Time Saving 1	2nd POCT hs-cTn	2nd Main Lab hs-cTn	Time Saving 2
Median	18	76	54	197	256	49
Q1	11	63	44	188	232	39
Q3	30	94	69	211	273	67
	POCT vs. Main lab arrival	*p* < 0.001		POCT vs. Main lab 2nd	*p* < 0.001	

Abbreviations: POCT: point-of-care testing; hs-cTn: high-sensitivity cardiac troponin.

## Data Availability

All data generated or analyzed during this study are included in this published article.
